# Vitamin A, Cancer Treatment and Prevention: The New Role of Cellular Retinol Binding Proteins

**DOI:** 10.1155/2015/624627

**Published:** 2015-03-24

**Authors:** Elena Doldo, Gaetana Costanza, Sara Agostinelli, Chiara Tarquini, Amedeo Ferlosio, Gaetano Arcuri, Daniela Passeri, Maria Giovanna Scioli, Augusto Orlandi

**Affiliations:** Anatomic Pathology, Department of Biomedicine and Prevention, Tor Vergata University, Via Montpellier, 00133 Rome, Italy

## Abstract

Retinol and vitamin A derivatives influence cell differentiation, proliferation, and apoptosis and play an important physiologic role in a wide range of biological processes. Retinol is obtained from foods of animal origin. Retinol derivatives are fundamental for vision, while retinoic acid is essential for skin and bone growth. Intracellular retinoid bioavailability is regulated by the presence of specific cytoplasmic retinol and retinoic acid binding proteins (CRBPs and CRABPs). CRBP-1, the most diffuse CRBP isoform, is a small 15 KDa cytosolic protein widely expressed and evolutionarily conserved in many tissues. CRBP-1 acts as chaperone and regulates the uptake, subsequent esterification, and bioavailability of retinol. CRBP-1 plays a major role in wound healing and arterial tissue remodelling processes. In the last years, the role of CRBP-1-related retinoid signalling during cancer progression became object of several studies. CRBP-1 downregulation associates with a more malignant phenotype in breast, ovarian, and nasopharyngeal cancers. Reexpression of CRBP-1 increased retinol sensitivity and reduced viability of ovarian cancer cells *in vitro*. Further studies are needed to explore new therapeutic strategies aimed at restoring CRBP-1-mediated intracellular retinol trafficking and the meaning of CRBP-1 expression in cancer patients' screening for a more personalized and efficacy retinoid therapy.

## 1. Retinol and Derivatives

### 1.1. Metabolism of Retinol and Its Derivatives

**Figure 1:**
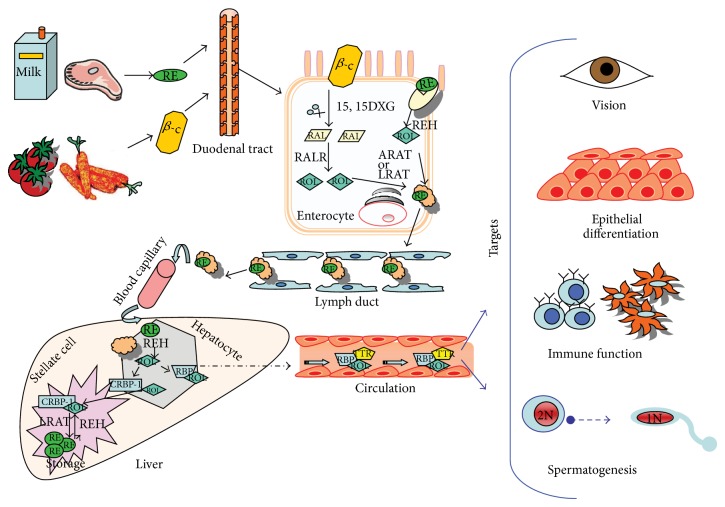
Absorption, transport and distribution of dietary retinoids.

Vitamin A can be acquired from the diet either as preformed vitamin A (primarily as retinyl ester, retinol, and in much smaller amount as retinoic acid) or provitamin A carotenoids (Figure 1). Dietary retinyl esters are converted to retinol within the lumen of the small intestine or the intestinal mucosa and then reesterified to form retinyl ester (RE) within the enterocyte [1]. Provitamin A carotenoids, absorbed by the mucosal cells, are converted first to retinaldehyde and then to retinol [1]. After secretion of the nascent chylomicrons into the lymphatic system, the bulk of dietary vitamin A is taken up by hepatocytes and hydrolyzed again. The free retinol binds the epididymal retinoic acid binding protein (ERABP) and the retinol binding protein (RBP) [2] and into plasma transthyretin. Free retinol can be transferred to hepatic stellate cells for storage. Hepatocytes and hepatic stellate cells are very rich in retinyl ester hydrolases and in cellular retinol binding protein type 1 (CRBP-1). CRBP-1 is necessary to solubilize retinol in the aqueous environment of the cell [1].

### 1.2. Intracellular Trafficking of Retinoids

**Figure 2:**
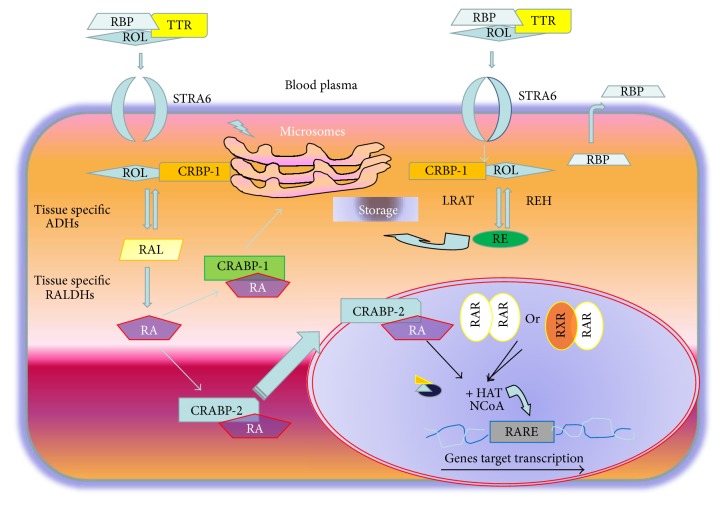
Intracellular retinoid pathways.

A cell-surface receptor named* stimulated by* retinoic acid 6 (STRA6) mediates vitamin A uptake from RBP [3]. Intracellular retinoid bioavailability is regulated by the presence of specific cytoplasmic retinol and retinoic acid binding proteins, CRBPs and CRABPs (Figure 2). In the cytoplasm vitamin A and derivatives are bound to cytoplasmic proteins: cellular retinol binding proteins (CRBPs) which comprised four isoforms, CRBP-1 and CRBP-2 and CRBP-3 and CRBP-4. CRBP-1, are the most represented isoform in many tissues. Cellular retinoic acid binding proteins (CRABPs) comprised two isoforms, CRABP-1 and CRABP-2. CRBPs specifically bind retinol, while CRABPs and well-characterized members of the fatty acid binding proteins (FABPs) bind retinoic acid (RA). These proteins control the availability of ligands and determine the physiological response of cells and tissues to vitamin A [4]. Cellular retinoic acid binding proteins may regulate the interactions between retinoic acids and their nuclear receptors by regulating the concentration of present retinoic acids [5]. Retinoids can activate gene expression by specific nuclear retinoid acid receptors. Two distinct classes of nuclear proteins, the retinoic acid receptors (RARs), and the retinoid X receptors (RXRs) have been identified. Each class consists of*α*, *β*, and *γ* subtypes. RARs and RXRs form either homodimers or heterodimers and function as transacting nuclear transcriptional factors [6]. RAR can be activated by both all-trans and 9-cis RA, whereas RXR is only activated by 9-cis-RA. Heterodimerization of retinoid receptors is essential for the biological activity, although it should be noted that RXRs can heterodimerize with numerous other non-RA associated nuclear receptors to mediate alternative signaling pathways. In the presence of retinoids, nuclear receptors bind to their respective response elements RAREs and RXREs in regulatory regions of target genes and modulate gene transcription [7].

### 1.3. Retinoids, Tissue Development, and Differentiation

Vitamin A and its derivatives are essential for biological processes such as vision, immune function, reproduction, maintenance of epithelial tissue, and differentiation. Vitamin A deficiency causes different pathological consequences such as night blindness, loss of vision, retardation, shortening and thickening of bones, atrophy of the testes, foetal reabsorption, and immunodeficiency [8]. Instead, an excess of vitamin A can cause teratogenic effects including major alterations in organogenesis [9]. So vitamin A is very important during embryonic development and adult tissue regeneration [10]. The best characterized bioactive metabolites of vitamin A are 11-cis retinal and all-trans retinoic acid (ATRA). The 11-cis retinal metabolite mediates photoreception by acting as the visual chromophore. Most of the nonvisual functions of vitamin A are mediated by ATRA, which regulates the expression of specific subsets of genes within target tissues via nuclear receptors. ATRA can be easily detected in many adult and embryonic tissues [11, 12]. A peculiar kind of retinol-storing cell can be found also in the lung [13]. These cells designated lipid interstitial cells are located in the alveolar wall and release ATRA synthesized from retinol into serum-free [13]. The release of ATRA induces changes in gene expression that initiate the formation of alveoli. It is noteworthy that, during alveolar formation, an increased expression of CRBP-1 is found in the pulmonary microvascular endothelial cells [13]. RA is not synthesized during all stages of development, but his production is restricted in a unique spatiotemporal pattern [14]. RA is expressed in a specific anterior-posterior pattern and its expression becomes more restricted during organogenesis [14]. In the adult RA levels are kept by retinol esterification through CRBP-1 in hepatic stellate cells [15]. Moreover, studies with RAR knockout mice indicate that RAR-*α* contributes to the regulation of alveolus formation after, but not during, the perinatal period [16], whereas RAR-*β* is an inhibitor of alveolus formation during, but not after, the perinatal period [17]. These receptors are important as developmental period-specific regulators of alveolus formation [13]. It has been known for more than 60 years that retinoids are involved in regulating the differentiation of various epithelia, promoting the formation of secretory epithelia and inhibiting the formation of highly keratinized, cornified epithelia. They also play a significant role in the control of endometrial growth and differentiation [18]. Given the dramatic shifts in differentiation that occurs in the rodent cervical epithelium from an early secretory phase to a later keratinizing phase, it is likely that retinoids play an important role in modulating the hormonal regulation of cellular differentiation in this tissue; the expression of retinoid binding proteins and nuclear receptors in a given tissue or cell type should be predictive of retinoid responsiveness itself. During pregnancy, in addition, spatial variations in the presence of cellular retinoid binding proteins have been observed, together with differences in patterns of expression between the functionally distinct upper and lower uterine segments [19]. Temporal variations were found with patterns of expression evolving through the three trimesters of pregnancy. This could be important for the regulation of myometrial proliferation* in vivo*. Moreover, RA is known to inhibit the binding of activator protein 1 to the cyclic AMP response element in the COX-2 promoter [20], so inhibiting phorbol-ester mediated induction of COX-2 gene transcription, whose intracellular levels consequently fall, reducing also prostaglandin production [20]. This process in human myometrium during pregnancy means that RA could contribute to the maintenance of uterine quiescence [19].

### 1.4. Retinoids and Vascular Pathology

Retinoids target numerous genes implicated in the pathological processes after vascular injury. The latter involves proliferation, differentiation, and migration of medial smooth muscle cells (SMCs) as well as matrix deposition [21–23]. Vascular postinjury, dedifferentiation, growth, and migration result in the formation of a neointima [24]. SMCs migrate to the luminal layer of the arterial wall after endothelial injury. Here growth factors and cytokines induce SMCs to enter into active phases of cell cycle [25]. SMCs proliferation plays also a critical role in the development of restenosis after coronary angioplasty and in the development of fatty streaks to the atherosclerotic plaques [23, 26]. A study shows two kinds of retinol metabolism in vascular SMCs of different phenotypes [24] and, more specifically, an increased uptake of retinol in intimal SMCs compared to medial SMCs, together with an increased expression of the retinoid metabolizing enzymes, such as retinol and retinal dehydrogenase. Consequently, an increased production of RA is found in intimal cells while a higher level of CYP26A1, the retinoic acid catabolizing enzyme, is observed in medial SMCs. Intimal SMCs show a dose- and time-dependent growth inhibition when treated with retinol in contrast to medial SMCs, in which retinol has a mitogenic effect. Moreover, intimal SMCs are more sensitive to RA than medial SMCs [22] and propyonil-L-carnitine increased apoptosis [27, 28]. In fact, systemic administration of RA significantly reduces arterial intimal thickening (IT) after endothelial injury* in vivo* [29] and induces apoptosis of CRBP-1 expressing intimal cells* in vitro*, but not of normal media SMCs [22]. Both* in vivo* and* in vitro* studies show that neointimal SMCs also display proinflammatory properties, such as higher expression of tumour necrosis factor-*α* (TNF-*α*) [30], so contributing to atherosclerotic process and restenosis. The different retinol metabolism in the two SMCs phenotypes supports the role of retinoids in preventing vascular proliferative disorders [31].

### 1.5. Retinoids and Cancer Therapy

Epidemiological studies have suggested an inverse correlation between cancer development and dietary consumption of vitamin A. Pharmacological concentrations of vitamin A decrease the incidence of chemically induced experimental tumours [32]. Natural and synthetic retinoids have been demonstrated to inhibit the growth and the development of different types of tumours, including skin, breast, oral cavity, lung, hepatic, gastrointestinal, prostatic, and bladder cancers [33–37]. Moreover, the addition of RA or synthetic retinoids to human cancer cell lines or human tumour xenografts in nude mice result in growth arrest, apoptosis, or differentiation [32]. It is noteworthy that natural retinoids act as chemotherapeutic agents for the treatment of acute promyelocytic leukemia (APL). APL is a subset of acute myeloid leukaemia characterized by uncontrolled expansion of leukemia blast cells, blocked at promyelocytic stage of hematopoiesis in the bone marrow [38]. It is characterized by a reciprocal translocation between the long arms of chromosomes 15 and 17 [*t*(15; 17)] [39–42]. This aberration leads to the fusion between the promyelocytic leukemia (PML) gene located on chromosome 15q21, normally responsible for the formation of the nuclear bodies and regulation of the stem cells self-renewal [43, 44] and the (RAR-*α*) gene from the chromosome 17q21, with the formation of a chimeric oncoprotein PML-RARA [41, 42, 45]. The fusion transcript is detectable in more than 95% of APL patients with *t*(15; 17) and acts as an oncogene, causing an enhanced DNA hypermethylation, proliferation, and inhibition of terminal differentiation of hematopoietic cells [46, 47]. ATRA-induced degradation of the fusion product is a basic therapeutic mechanism in APL cells [41, 48]. Recent results reveal several mechanisms leading to the destruction of the fusion oncogene, such as ubiquitination [49], sumoylation [50], or autophagy [51]. High concentrations of ATRA induce postmaturation apoptosis of APL-blasts through the induction of the tumour-selective death ligand TNF-related apoptosis-inducing ligand [52]. Effectiveness of ATRA in the treatment of APL observed in more than 90% of the patients leading to the complete remission [53–55]. Moreover, in animal models several studies establish an inhibitory role of retinoids in breast cancer. It was reported a 52% reduction in the incidence of mammary cancer in animals treated with retinyl acetate [56].* In vitro* studies show that retinoids, in particular 9-cis-RA, inhibit the growth of oestrogen receptor- (ER-) positive through blocking cell cycle [57], but not ER-negative human breast cancer cells [25, 58]; ER-negative cells have been demonstrated to express lower RAR-*β* levels compared to their ER-positive matched cells [59] and they exhibit retinol-induced growth inhibition when transfected with RAR-*β* [60]. Preclinical studies demonstrate that ATRA induces cell cycle and proliferation arrest in breast cancer cells [61] through the modulation of cyclin-dependent kinase inhibitors p21^WAF1^ and p27^KIP1^, with dephosphorylation of retinoblastoma protein [62]. Primary brain tumours are among the top ten causes of cancer-related deaths in the USA. Gliomas, in particular, may result from an imbalance in retinoid receptor expression initiated by environmental factors that increase the endogenous production of RA in the glial cells [63]. It is proposed that this imbalance is characterized by excessive expression of RAR-*α* and reduced expression of RAR-*β*. The combined use of RAR-*α* antagonist and RAR-*β* agonist is suggested as potential new treatment strategy for gliomas, possibly even at a late stage of the disease. According to this hypothesis, the RAR-*α* antagonist would be expected to inhibit RAR-*α*-induced gliomas, while the RAR-*β* agonist would suppress tumour growth and possibly contribute to the regeneration of normal glia [63]. Moreover, vitamin A reduces the induction of carcinoma of the stomach by polycyclic hydrocarbons [64] and vitamin A-deficient rats are more susceptible to induction of colon tumours by aflatoxin B than normal animals [65]. The combination treatment of histone deacetylase inhibitors SL142 or SL325 with retinoic acids exerts a significant antitumour activity and is a promising therapeutic candidate to treat human lung cancer and show antitumour effect in neuroblastoma [66]. While synthetic retinoids are generally promising for cancer treatment, only few of them are FDA-approved or currently undergoing clinical trials for cancer therapy. Preclinical studies show that synthetic retinoids inhibit human cancer growth. Fenretinide (4-HPR) is one of the most promising clinically tested retinoids. 4-HPR demonstrated a significant cytotoxic activity of tumour cells through the induction of apoptotic and nonapoptotic cell death [67] in breast [68, 69], prostate, bladder, skin [52, 70–74], colon-rectal [75], head and neck [76], ovarian cancers [77, 78], both small cell and non-small cell lung cancer [79–81], neuroblastoma [82–84], and leukaemia cell lines [85, 86]. 4-HPR has activity against tumours by generating reactive oxygen species [76, 87, 88], increasing dihydroceramide production [87, 89–91] and natural killer cell activity [92, 93] and inhibiting angiogenesis [89, 94]. TAC-101 has shown efficacy in inhibiting liver tumour growth in particular of hepatocellular carcinoma [95, 96]. Synthetic retinoids, approved from FDA for dermatological purposes, have a potential antitumour activity. Bexarotene is a retinoid that specifically binds retinoid X receptors and has numerous effects on cellular growth and differentiation. It is approved for the treatment of cutaneous T-cell lymphoma both topically and systemically [97, 98]. A preliminary clinical experience suggests tazarotene, a new acetylenic retinoid, as an effective alternative topical treatment of basal cell carcinomas. Tazarotene demonstrated a good efficacy in the therapy of basal cell carcinoma [99, 100].

## 2. Cellular Retinol Binding Proteins

### 2.1. The Specific Role of CRBP-1 in Retinol Metabolism

CRBP-1 is a small cytosolic protein of 15 KDa. CRBP-1 is widely expressed and evolutionarily conserved in many tissues and acts as chaperone protein that regulates the uptake and subsequent esterification of retinol and its bioavailability. CRBP-1 gene is located on 3q21 chromosome. CRBP-1 is required for the efficient synthesis of ATRA [101]. CRBP-1 binds retinol and interacts with enzymes involved in esterification of retinol with long chain fatty acids and in hydrolysis of retinyl esters into retinol.* In vitro*, CRBP-1 also channels either retinol towards microsomal dehydrogenases that catalyze the oxidation of retinol into RAL or its addressing towards cytosolic dehydrogenases for oxidation into RA. Several microsomal enzymes, termed retinol dehydrogenases types I, II, and III, have high specificity for CRBP-1-bound retinol. Retinal dehydrogenases oxidize CRBP-1-bound retinal [102]. Retinal-CRBP-1 formed through oxidation of retinol-CRBP-1 by the microsomal retinol dehydrogenase might be directly oxidized to RA by retinal dehydrogenases. CRBP-1 may also delivery retinol to newly synthesized RBP for secretion from the liver into the circulation. In the mouse, CRBP-1 deficiency decreases the capacity of hepatic stellate cells to take up incoming retinol and to maintain RE stores, because of an accelerated rate of RE turnover, indicating that CRBP-1 is essential for efficient retinol storage during postnatal life [4].

### 2.2. Role of CRBP-1 during Development

It has been shown that a lack or an excess of RA during embryonic development results in congenital abnormalities. As a matter of fact, RA regulates genes that specify body axis pattern and may help to program limb formation in the developing embryo. In mature vertebrates, RA maintains epithelial tissues, contributes to bone remodelling and sustains reproductive processes, such as the oestrous cycle, spermatogenesis, and placental growth [103]. As RA synthesis previously seemed to involve intricate metabolic pathways, carried out by multiple enzymes recognizing both liganded (holo-CRBP-1) and nonliganded forms (apo-CRBP-1) of CRBP-1, the role of this protein in organ development has deserved to be deepened. So in mice, the absence of CRBP-1 does not show to be life-threatening during development, at least under conditions of maternal vitamin A dietary sufficiency [4]. However, retinol and RE contents were lower in CRBP-1−/− embryos and foetuses than in WT [4]. Researchers hypothesized that this depends on a reduced transfer of retinol from the maternal circulation to the foetus through the placenta, where it is normally expressed, as CRBP-1 enhances retinol uptake by placental membranes* in vitro* [104]. It could also depend on an altered retinol uptake by the foetal cells themselves [4]. As a matter of fact, tritiated retinol, administered to pregnant WT mice, specifically accumulates in regions of the embryo expressing CRBP-1 [105]. These lower ER contents could finally depend on a reduced RE half-life, as in adult liver [106] or to an impaired delivery of retinol to specific enzymes for esterification. The normal embryonic development of CRBP-1 null mice suggests that, even though involved in RA homeostasis, CRBP-1 is not critically required for development as its ablation does not change, at least under conditions of maternal vitamin A sufficiency, the expression of RA-target genes during prenatal life. Even if no embryonic, foetal, or placental abnormality could be detected in CRBP-1-null mice (indicating the dispensability of this protein during intrauterine development), CRBP-1-null mice, fully exhausted their RE stores within 5 months and developed abnormalities characteristic of postnatal hypovitaminosis A, as also described in rats [107], and, namely, consisted in vision defects, testicular degeneration, and squamous keratinizing metaplasia. The decrease capacity to store incoming retinol in CRBP-1 null liver could depend on a higher hydrolysis of hepatic stores, due to a decrease of RE half-life and higher amounts of retinol in blood. This hydrolysis could be attributed to an impaired delivery of retinol to esterifying enzymes, above all LRAT, that requires CRBP-1-bound retinol as a substrate [108]. The morphological appearance of CRBP-1 null testes after 14 weeks under the vitamin A deficient diet represents a clear phenocopy of the RAR-*α*-null mutation, while the squamous keratinizing metaplasia observed in CRBP-1-null mice fed with the vitamin A deficient diet is similar to those observed in old adult mice lacking RAR-*γ* [109].

### 2.3. Expression of CRBP-1 in Normal and Adult Tissues

CRBP-1 expression has been studied at histological distinct stages of the rat oestrus cycle. CRBP-1 was detected during the early proliferative phase and after the formation of a mucinous cell layer during proestrus [18]. CRBP-1 expression closely follows the state of differentiation of the cervical epithelial cells. High levels were found in both columnar cells and incompletely differentiated epithelial cells [110]. Decreased CRBP-1 expression coincides with the loss of retinol responsiveness in rat cervical epithelial cells [18], whileCRBP-1 expression was higher in the atrophied epithelium and both CRBP-1 mRNA and protein levels decreased when animals were treated with oestrogen. During proestrus high CRBP-1 expression indicates that high local levels of retinol or retinoic acid are either directly required for the formation of these secretory cells or indirectly required to inhibit keratinization. CRBP-1 levels subsequently decline and the epithelium extensively keratinizes, this might limit retinol uptake and metabolism [18]. It is worth noting that the pattern of CRBP-1 expression in the human endocervical epithelium is identical to that reported for the rat [111]. Moreover, in human, nonpregnant myometrium CRBP-1 is easily available, together with the CRABP proteins, thus suggesting a role for ATRA in the control of myometrial proliferation* in vivo*, but CRBP-1 is downregulated in both the upper and the lower uterine segments during the first and the second trimester of pregnancy [19]. By the end of the third trimester, CRBP-1 is upregulated in upper segment myometrium, together with CRABP-2 [19]. CRBP-1 expression characterizes endometrial stromal cells at eutopic and ectopic sites and appears to be more specific than CD10 [112]. The level of CRBP-1 varies in intensity according to hormonal variations, with an increase during the secretory phase compared with those of proliferative endometrium. The highest CRBP-1 immunodetection was observed in predecidual and decidual tissue. CRBP-1 expression in the endometrial stroma was similar to that of CD10. Thus, immunodetection of CRBP-1 may help to elucidate the physiopathological changes which occur in endometrial stroma and can also be applied as an adjuvant stromal marker [112].

### 2.4. Role of CRBP-1 in Tissue Remodelling

One of the key events in the wound repair process is the infiltration of fibroblasts into the damaged area where they proliferate and differentiate into myofibroblasts expressing features of SMCs. These fibroblasts present in the granulation tissue originate from subcutaneous tissue fibroblasts [113]. CRBP-1 expression strongly increased during wound healing in rat skin, suggesting that it plays a role in the evolution of granulation tissue [114]. Liver myofibroblasts derived from hepatic stellate cells or from portal fibroblasts express CRBP-1. HSC express CRBP-1 both in normal liver and during liver fibrosis, as these cells are involved in myofibroblast differentiation [115]. Aberrant CRBP-1 expression has shown to be accompanied by parallel variations of*α*-smooth muscle actin. The*α*-smooth muscle actin is mainly controlled by TGF-*β*1, a fibrogenic mediator involved in both myofibroblast differentiation and extracellular matrix deposition. Surprisingly, TGF-*β*1 induces CRBP-1 gene and protein expression in primary cultures of HSC and in portal fibroblasts. The myofibroblast differentiation of HSC is associated with a decrease in total hepatic levels of retinyl palmitate [116], the predominant storage form of vitamin A in rat HSC, whereas levels of retinol are increased together with CRBP-1 levels [117]. So, it is possible that the stable expression of CRBP-1 in HSC during liver fibrosis is under the control of free retinol level [115]. The pattern of CRBP-1 expression in portal fibroblasts is similar to that observed in subcutaneous fibroblasts or in arterial SMCs that, after injury, rapidly acquire CRBP-1 expression [118]. The expression of CRBP-1 in arterial SMC during arterial repair, in myofibroblasts during skin wound healing and in portal fibroblasts during liver reaction to bile duct ligation, strongly supports that CRBP-1 plays a role in multiple tissue repair phenomena [118]. As a matter of fact, distinct rat aortic SMC subpopulations and clones express CRBP-1 and a CRBP-1-containing SMC subpopulation appears transiently* in vivo* during the evolution of the experimental aortic IT produced by endothelial injury. CRBP-1 expression in cultured SMCs has shown to be regulated by ATRA and retinol. Moreover, the presence of CRBP-1 in cultured SMCs appears to be correlated with the expression of cytokeratin 8 [119]. CRBP-1 has also shown to be constitutively expressed in aortic endothelial cells and, occasionally, in SMCs of the normal media of adult and old rats but not in newborn rats [118]. It is conceivable that the scattered CRBP-1-positive SMCs of the media participate in the formation of IT after endothelial injury and a proportion of CRBP-1-negative SMCs becomes positive, as the large majority of the IT SMCs express CRBP-1 at 7 days. These results suggest that a subset of medial SMCs becomes rapidly CRBP-1 positive after injury, undergoes replication during the early phases of IT development, and then disappears, possibly through apoptosis, when reendothelialization takes place. So, CRBP-1 is not simply a marker of SMC activation but participates in this process [118].

### 2.5. CRBP-1 and Cancer Progression: New Therapeutic Perspectives

**Figure 3:**
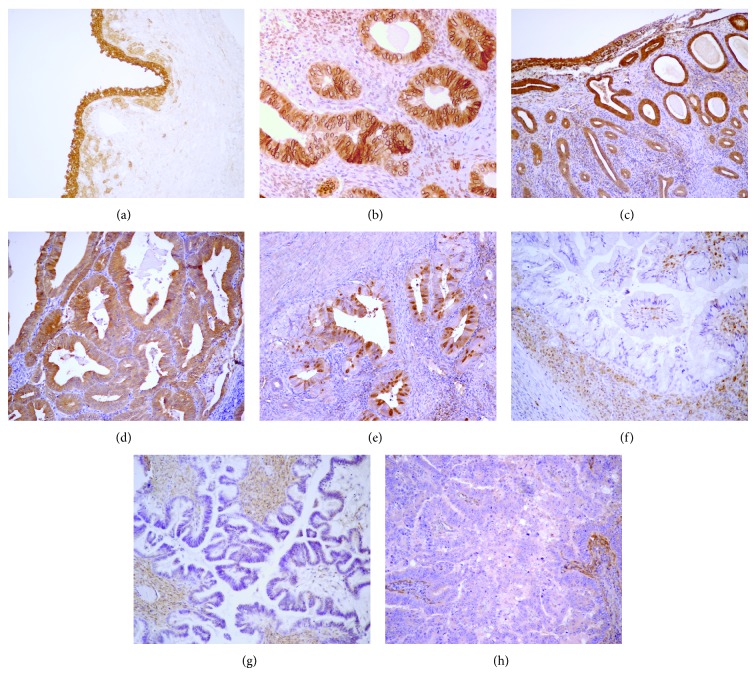
Immunohistochemical expression of CRBP-1 in normal and neoplastic female reproductive system tissues. (a) CRBP-1 is strongly expressed in normal endocervical epithelial cells, (b) proliferative and (c) atrophic endometrial glandular cells. (d) Well-differentiated (G1) endometrial carcinoma cells show strong and diffuse CRBP-1 positive cells compared to (e) lower and focal CRBP-1 expression in moderately (G2) carcinoma. In the ovary, CRBP-1 is not expressed in (f) mucinous, (g) serous borderline, and (h) well-differentiated (G3) serous carcinoma. Original magnification: (a), (c)–(h) 100x; (b) 200x.

Because of the complexity of vitamin A metabolism, the precise role of CRBP-1 in retinoid signaling remains controversial, despite numerous studies conducted over the past three decades on its binding properties, three-dimensional structure, tissue localization, regulation of expression, involvement in retinal metabolism, and null mutation [120]. The function of the CRBP-1 gene in controlling the cell bioavailability of vitamin A suggests that it can have a special relevance in the inhibition of early steps of cancer transformation. Nevertheless, in human cancer, the presence and role of the specific binding proteins for retinol and RA have not been extensively investigated. Downregulation or loss of CRBP-1 expression occurs in a series of tumors: breast, ovarian, endometrial, prostate, renal cancer, astrocytic gliomas [112, 121, 122], cervical cancer, larynx cancer, nasopharyngeal carcinoma, lymphoma, and gastrointestinal carcinomas [123]. Furthermore, the CpG island hypermethylation of CRBP-1 is responsible for its inactivation in some cancer cell [124–127]. So, epigenetic disruption of CRBP-1 is a common event in human cancer and may have important implications for cancer prevention and retinoid therapy. What are the biological consequences of the methylation-mediated silencing of CRBP-1? The loss of CRBP-1 may compromise retinoic acid metabolism by reducing retinol transport and blocking the formation of retinyl esters and RAR activity, leading to loss of cellular differentiation and tumor progression [123, 128–130]. CRBP-1 downregulation was observed in stage I as well as in stage II and III patients and also reported in ovarian cancer precursors, suggesting that interruption of CRBP-1 signaling may occur at all stages of cancer progression [120]. Several studies highlighted the role of CRBP-1 signaling in cancer progression during the last years [131], but the mechanisms by which it affects carcinogenesis are far from being fully elucidated. Uterine and gastrointestinal leiomyosarcomas express high levels of CRBP-1, whereas its expression is weak in leiomyoma, symplastic leiomyoma, borderline tumours, and nontumour smooth muscle tissue [27]. Accumulation of CRBP-1 in leiomyosarcoma likely supports the conversion of retinol into RA and its biological effects through RAREs induction, which influence the expression of many genes through the interaction with RAREs regions, inducing the increase of CRBP-1 expression in epithelioid SMCs as well as fibroblasts [29]. These results support the possibility that the expression of CRBP-1 represents a target for pharmacological strategies aimed at influencing sarcoma growth through the control of RA availability. About 30% of colorectal cancers did not present any apparent lesion in the retinoid pathway and this subset of tumors may be extremely sensitive to treatment with retinoids [123]. With regard to liver pathologies, CRBP-1 expression is downregulated in almost all hepatocellular carcinoma specimens [132]. Some studies show that CRBP-1 is strongly expressed in the cytoplasm of hepatic stellate cells in normal liver and in myofibroblasts, with only low CRBP-1 levels in hepatocytes. Patients with high CRBP-1 expression in myofibroblasts show a significantly higher 2-year survival as compared with patients with low CRBP-1 expression [132]. The loss of CRBP-1 expression in intratumoral myofibroblasts likely disturbs retinoid homeostasis, thereby potentially leading to more aggressive liver tumour growth. These studies also highlight a strong positive association between nuclear CRBP-1 expression and nuclear *β*-catenin accumulation [132, 133]. This cotransactivation complex exerts several regulatory effects on a growing number of downstream target genes, many of which have been implicated in tumorigenesis [134]. In a case-controlled CRBP-1 study in gynaecological cancers, significant differences were found between the concentrations of CRBP-1 in the dysplastic cervical lesions and the normal cervix [122]. CRBP-1 was detected at a reduced level in the carcinoma poorly differentiated of endometrium, ovary (Figure 3), and breast compared with normal tissue aliquots. CRBP-1 immunodetection in simple hyperplasia was weakly and similar to that of proliferative endometrial cells and increased in atypical hyperplasia and in G1 endometrioid carcinomas [122]. Importantly, a progressive decrease of CRBP-1 immunoreactivity was observed in less differentiated endometrial tumours [122]. The striking overall difference in the expression of CRBP-1 in type I and type II endometrial carcinomas reflects the differences in their risk factors and molecular pathogenesis [122]. The low or absent CRBP-1 expression in serous and clear cell carcinomas further supports the presence of distinct molecular carcinogenetic pathways [122].

**Figure 4:**
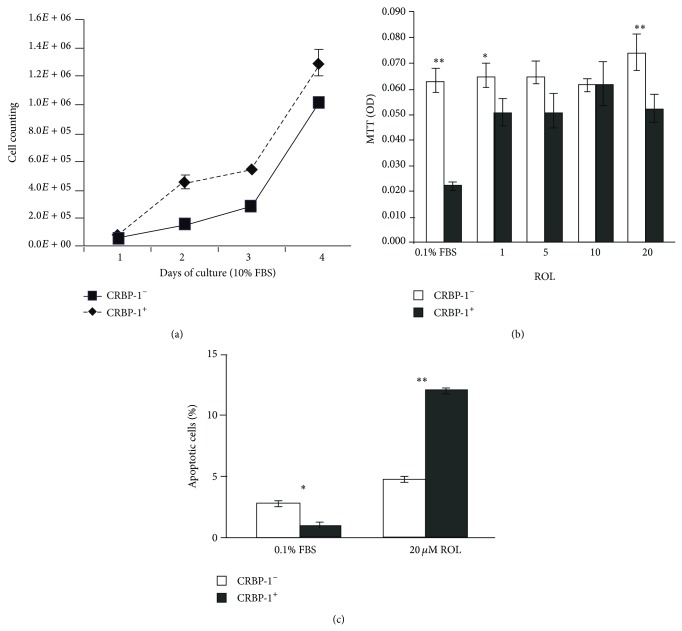
CRBP-1^+^ expression influences retinoid-induced A2780 cancer cell viability. (a) Viability of transfected CRBP-1 (CRBP-1^+^) A2780 cells increased after 4 days in the presence of 10% FBS compared to empty transfected (CRBP-1^−^) A2780 cells. (b) Retinol induces a reduction of viability in CRBP-1 A2780 cells after 24 h of different ATRA treatments (c) Flow cytometry analysis of Annexin V/PI apoptotic assay shows a higher percentage of dying cells in CRBP-1^+^ after 2 h of 20 *µ*M retinol treatment compared to CRBP-1^−^ A2780 cells. Values are expressed as means ± SEM of three different experiments; ^*^ *P* < 0.05, ^**^ *P* < 0.01.

It has been documented that CRBP-1 upregulation exerts a direct antitranscriptional effect through the binding of CRBP-1 promoter to RAR-*α* [135].* In vitro* studies confirmed the downregulation of RAR-*α* and RAR-*γ* mRNA levels in CRBP-1-stable-transfected ovarian cancer cells [127]. Abnormal RAR-*α* upregulation characterizes other malignancies, such as lung cancer and metastatic melanoma [121]. Similarly, RAR-*γ* upregulation is reported to be prooncogenic and to support the growth/progression of mammary tumours [136]. So, CRBP-1-mediated reactivation of retinol pathways could improve the efficacy of adjuvant retinoid chemotherapy, likely increasing apoptotic susceptibility [127, 137–139] (Figure 4).

Furthermore, CRBP-1 induces a reduced transcription level and activity of PI3K/Akt pathway, in particular, Akt1 [127]. Although Akt contributes to maintain differentiation of stem cells [140], aberrant pAkt activation sustains cancer progression [127]. CRBP-1 signaling restoration in A2780 ovarian cells induces the downregulation of specific cell proliferation and survival genes STAT1, STAT5, and JUN [127, 141] and downregulation of pErk. The latter is critically involved in the regulation of cell proliferation and survival and its upregulation promotes cancer proliferation, survival, and metastasis [142]. CRBP-1 can have a function independent of its retinol binding ability; in this regard, CRBP-1 also functions in mammary epithelial cell inhibition of the phosphatidylinositol 3-kinase/Akt survival pathway and suppresses anchorage independent growth [121]. Interestingly, somatic CRBP-1 silencing, induced by inoculation of athymic mice with MTSV cells, an SV40-immortalized human mammary epithelial cell line, transfected with empty vector (MTSV^vector^) or CRBP-1 (MTSV^CRBP-1^), has shown to prevent tumor cells from taking up, storing, and using retinol* in vivo* [137]. These* in vivo* data suggest that somatic CRBP-1 loss results in a local deficit in vitamin A storage and metabolism, with important consequences for the affected tissues.

Reintroduction of CRBP-1 signaling reduced tumorigenicity both* in vivo* and* in vitro*, inducing downregulation of survival and proliferative gene pathways and increases retinoid-mediated apoptosis [127, 137, 143]. So, CRBP-1 can represent a potential target for therapeutic strategies aimed at arresting cancer cell growth and tumour progression by increasing intracellular retinol bioavailability [127, 137]. In addition, the presence of detectable CRBP-1 level in a subset of ovarian cancers [127] suggests screening of its expression for a more efficacy and personalized adjuvant retinoid-mediated therapy.

## 3. Conclusions

Vitamin A and derivatives comprise a group of natural and synthetic molecules, which regulate a variety of essential biological processes during normal development, maintained tissue homeostasis, and also mediate protection from diseases. Retinoids have many important and diverse functions throughout the body including roles in vision, regulation of cell proliferation and differentiation, growth of bone tissue, immune function, and activation of tumour suppressor genes. Genomic functions of the retinoids are mediated via their nuclear DNA-binding receptors, RARs, and RXRs, which regulate gene transcription through recruitment of corepressors and coactivators. Natural and synthetic retinoids have been used as potential chemotherapeutic or chemopreventive agents because of their differentiation, antiproliferative, proapoptotic, and antioxidant effects. The function of the CRBP-1 gene in controlling the availability of retinol to cells suggests that its product has special relevance to inhibition of early steps in transformation. CRBP-1 downregulation occurs in breast and ovarian tumors and compromises RAR activity, leading to loss of cellular differentiation and tumor progression. Furthermore, the CpG island hypermethylation of CRBP-1 is responsible for its inactivation in some cancer cell lines such as cervical, larynx, nasopharyngeal, and gastrointestinal carcinomas and lymphoma [123, 128–130]. So, the loss of CRBP-1 expression is a common event in human cancer that may have important implications for cancer prevention and treatment using retinoids. The possibility to reintroduce CRBP-1-mediated intracellular retinol trafficking in cancer cells can represent a potential tool in strategies aimed at counteracting cancer cell dedifferentiation and minor aggressiveness. Moreover, variability of CRBP-1 expression in some cancers also suggests screening of tumours in order to select patients potentially sensitive to adjuvant retinoid therapy.

## Abbreviations

STRA6: Stimulated by retinoic acid 6
RE: Retinyl ester
RBP: Retinol binding protein
REH: Retinyl ester hydrolases
CRBP-1: Cellular retinol binding protein 1
RARs: Retinoic acid receptors
RXRs: Retinoid X receptors
RAREs: Retinoic acid response elements
RXREs: Retinoid X receptor response elements
RA: Retinoic acid
ATRA: All-trans retinoic acid
CRABP-1 and CRABP-2: Cellular retinoic acid binding proteins 1 and 2
FABPs: Fatty acid binding proteins
TNF-*α*: Tumour necrosis factor-*α*
APL: Acute promyelocytic leukemia
PML: Promyelocytic leukaemia
ER: Oestrogen receptor
RAL: Retinaldehyde
SMC: Smooth muscle cells
TGF-*β*1: Transforming growth factor-*β*1
IT: Intimal thickening
LEF: Lymphoid enhancer factor
TCF: T-cell factor
4-HPR: Fenretinide.
